# Krüppel-like factor 4 (KLF4) regulates the miR-183~96~182 cluster under physiologic and pathologic conditions

**DOI:** 10.18632/oncotarget.15459

**Published:** 2017-02-17

**Authors:** Miguel F. Segura, Luz Jubierre, SiDe Li, Aroa Soriano, Lisa Koetz, Avital Gaziel-Sovran, Marc Masanas, Kevin Kleffman, John F. Dankert, Martin J Walsh, Eva Hernando

**Affiliations:** ^1^ Department of Pathology, New York University School of Medicine, New York, NY, USA; ^2^ Interdisciplinary Melanoma Cooperative Group, New York University Perlmutter Cancer Institute, NYU School of Medicine, New York, NY, USA; ^3^ Laboratory of Translational Research in Child and Adolescent Cancer, Vall d'Hebron Research Institute (VHIR)-UAB, Barcelona, Spain; ^4^ Departments of Structural and Chemical Biology, Genetics and Genomic Sciences and Pediatrics, Icahn School of Medicine at Mount Sinai, New York, NY, USA

**Keywords:** microRNA, embryonic stem cells, melanoma, miR-183∼96∼182 cluster, KLF4

## Abstract

MicroRNAs (miRNAs) are a class of endogenous non-coding small RNAs that post-transcriptionally control the translation and stability of target mRNAs in a sequence-dependent manner. MiRNAs are essential for key cellular processes including proliferation, differentiation, cell death and metabolism, among others. Consequently, alterations of miRNA expression contribute to developmental defects and a myriad of diseases.

The expression of miRNAs can be altered by several mechanisms including gene copy number alterations, aberrant DNA methylation, defects of the miRNA processing machinery or unscheduled expression of transcription factors. In this work, we sought to analyze the regulation of the miR-182 cluster, located at the 7q32 locus, which encodes three different miRNAs that are abundantly expressed in human embryonic stem cells and de-regulated in cancer. We have found that the Krüppel-like factor 4 (KLF4) directly regulates miR-182 cluster expression in human embryonic stem cells (hESCs) and in melanoma tumors, in which the miR-182 cluster is highly expressed and has a pro-metastatic role. Furthermore, higher KLF4 expression was found to be associated with metastatic progression and poor patient outcome. Loss of function experiments revealed that KLF4 is required for melanoma cell maintenance. These findings provide new insights into the regulation of the miR-182 cluster expression and new opportunities for therapeutic intervention in tumors in which the KLF4-miR-182 cluster axis is deregulated.

## INTRODUCTION

Non-coding RNAs including microRNAs (miRNAs) have emerged as key regulators of development and disease [1]. MiRNAs are a class of small non-coding RNAs that interfere with the translation and stability of coding messenger RNAs (mRNAs) in a sequence-specific manner [2]. Mature, biologically-active miRNAs are ∼22 nucleotides in length, and processed from longer primary transcripts by the RNAse enzymes Drosha and Dicer in the nucleus and cytoplasm, respectively [3]. Despite all the knowledge gathered in the last 15 years on their tissue-specific expression and functional consequences of their overexpression or silencing, relatively little is known on their own expression regulation. MicroRNA genes are distributed along the genome and can be intergenic, intronic (mirtrons), or exonic (in exons of coding or non-coding genes). They are found as single genes or organized in clusters, i.e, miRNAs located in the same genomic region, usually in less than 10Kb, and transcribed as a single transcriptional unit [4].

MiRNAs altered in cancer are often located in genomic regions that are prone to alterations including amplifications or deletions [5]. The high frequency of genomic alterations in miRNA loci was confirmed by high-resolution array-based genomic hybridization on 227 human ovarian cancer, breast cancer, and melanoma samples [6]. This study proved that miRNA expression strongly correlated with gene copy number. Our group showed that the melanoma metastasis promoter miR-182 is transcribed from the miR-183∼96∼182 cluster (hereafter referred as miR-182 cluster) in the 7q32 chromosomal region. This cluster is frequently overexpressed in melanoma tissues and cell lines and promotes migration *in vitro* and metastasis *in vivo* [7]. In around 50% of melanoma cell lines tested (7 out of 14 cell lines), miR-182 cluster overexpression was linked to amplification of the 7q31-34 locus, a region commonly amplified in melanoma, but the mechanism(s) underlying overexpression in the remaining cases remained unclear. This cluster has now been found to be deregulated in other tumors and disorders [8] in which genomic amplification seems not to be the main cause. Additionally, the miR-182 cluster is one of the most expressed in human embryonic stem cells (hESCs) [9, 10], suggesting a potential role in stem cell maintenance and regulation of differentiation.

In this study, we sought to analyze the transcriptional and epigenetic mechanisms that lead to miR-182 cluster expression in physiological (i.e. hESCs) and pathological (i.e. malignant melanoma) conditions. We describe the minimal promoter region that is sufficient to drive the expression of the miR-182 cluster. Among the several transcription factors predicted to bind this region, Krüppel-like factor 4 (KLF4) showed the highest trans-activation capacity, and expression correlation with miR-182 in melanoma cell lines without 7q amplification. Chromatin Immunoprecipitation (ChIP) analysis confirmed the physical binding of KLF4 to the miR-182 cluster promoter in melanoma cell lines but not in melanocytes, in which miR-182 is not expressed. Moreover, KLF4 also occupies the miR-182 cluster promoter in hESCs from which is gone during melanocyte differentiation. The mechanisms underlying the silencing of the miR-182 cluster in the melanocytic lineage involve histone deacetylation since the treatment of cells with histone deacetylase inhibitors restores miR-182 cluster expression. In sum, our study provides new insights into the regulation of this cluster in development and cancer and opens up new opportunities for therapeutic intervention in tumors in which the KLF4-miR-182 cluster is deregulated.

## RESULTS

### KLF4 is sufficient to activate the expression of the miR-182 cluster

**Figure 1:**
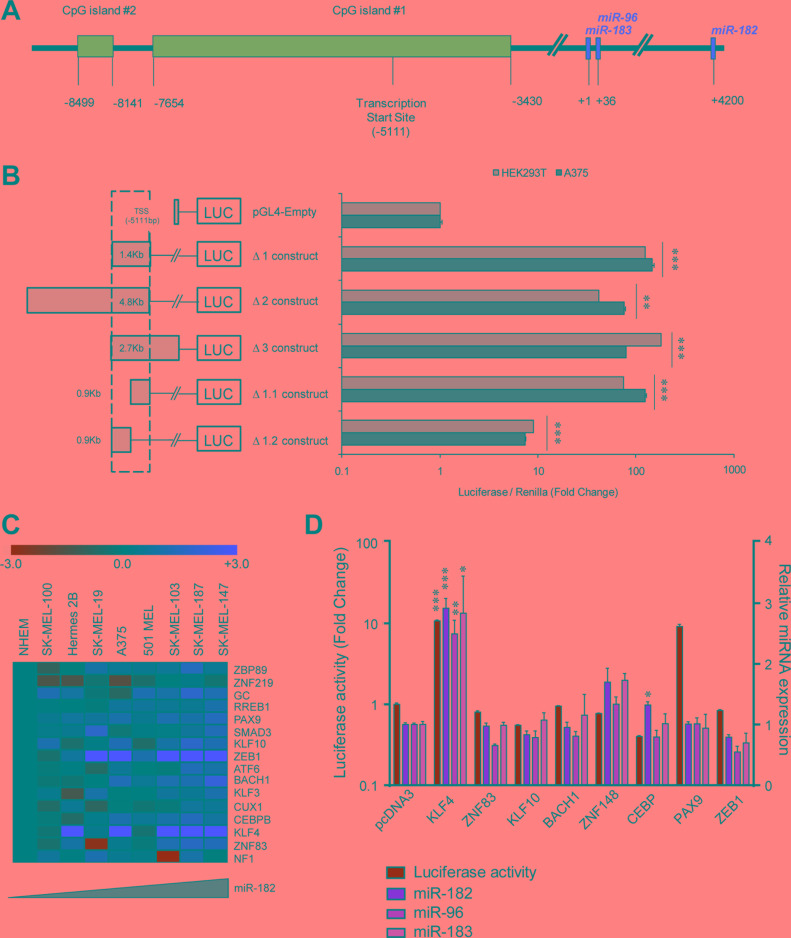
KLF4 regulates the miR-182 cluster promoter (**A**) Representative scheme of the miR-182∼96∼183 cluster locus. (**B**) Luciferase activity assay using reporter constructs flanking the predicted transcription start site (TSS) of the miR-182 cluster. (**C**) Heat map of the indicated transcription factors showing a positive correlation with miR-182 levels in melanoma cell lines without 7q amplification. (**D**) Luciferase activity assay of the miR-182 cluster promoter (green bars) and correlation with endogenous levels of miR-182 cluster (orange bars) measured by qPCR. *p < 0.05; **p < 0.01; ***p < 0.001.

In an attempt to delineate the functional promoter region of the miR-182 cluster, several luciferase reporter constructs flanking the predicted transcription start site [10] (TSS) (Figure 1A) were generated. Five different fragments of the two main CpG islands upstream the TSS were cloned upstream the luciferase reporter gene (Figure 1B, left). Luciferase activity assays were performed to determine the basal trans-activation capacity of those sequences in two different cell lines. We found that the Δ1.1 construct, which expands 0.9 Kb upstream of the TSS, retains the maximal luciferase activity levels across all the constructs tested (Figure 1B, right). *In silico* analysis (MatInspector, [11]) revealed that the Δ1.1 region contains 58 putative binding sites for 46 different transcription factors (TFs), with one or more binding sites conserved between the murine and human sequences (Supplementary Table 1). To narrow down the main potential regulator(s) of miR-182 cluster expression, we integrated mRNA microarray expression data for the 46 TFs with miR-182 levels measured by qPCR in a panel of melanoma cell lines without amplification of the 7q32 locus. Sixteen out of forty-six TFs revealed a positive correlation with miR-182 expression levels (Pearson's value > 0.5, Supplementary Table 2, Figure 1C). Eight candidate genes (KLF4, KLF10, ZEB1, ZNF83, ZNF148, CEBP, BACH1 and PAX9) were selected for further analysis based on their higher expression levels in melanoma compared to normal melanocytes, and individually tested as potential trans-activators of the minimal miR-182 cluster promoter in HEK293T cells. KLF4 and PAX9 transient ectopic expression significantly enhanced the basal luciferase activity of the Δ1.1 reporter construct, indicating that those TFs may modulate -directly or indirectly- the activity of the miR-182 cluster promoter (Figure 1D, green bars). However, in the same experimental samples, miR-182,-96, and -183 endogenous levels were only found upregulated in KLF4-transduced cells (Figure 1D, orange bars).

### KLF4 binds to the miR-182 cluster promoter and is necessary for miR-182 expression

**Figure 2:**
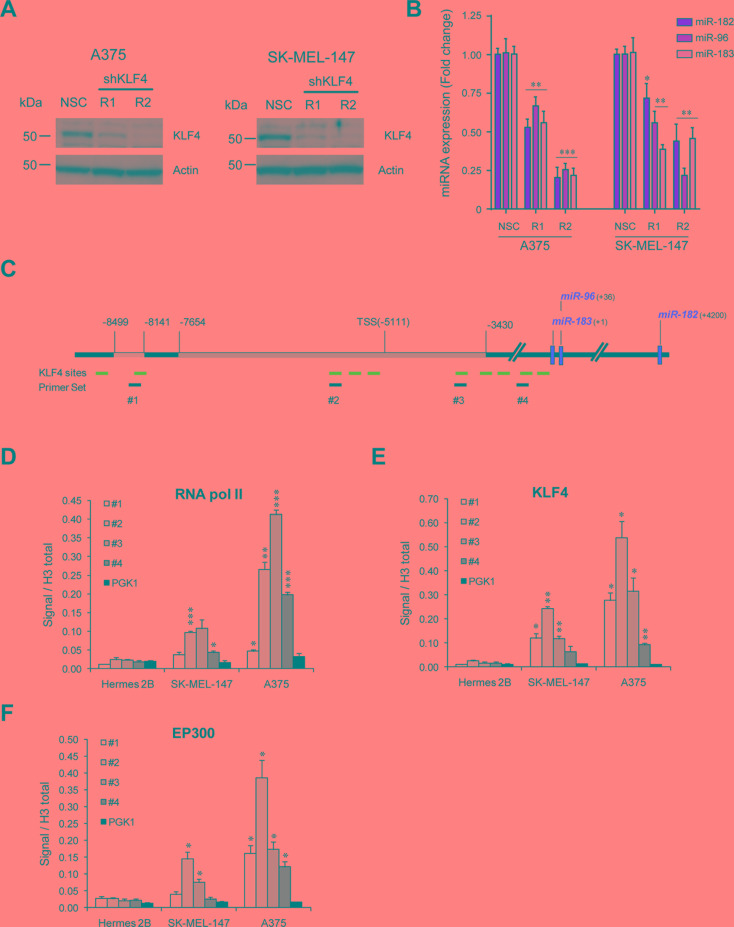
KLF4 controls miR-182 expression in melanoma cells (**A**) Western blot showing KLF4 knockdown 72 hours post-infection with the indicated shRNA lentiviral particles. (**B**) Expression levels of miR-182 cluster members upon KLF4 silencing in the indicated melanoma cell lines, measured by qPCR. (**C**), Representative scheme of the miR-182 cluster locus with KLF4 putative binding sites (in blue), and primer sets used in ChIP (black). (**D**–**F**), ChIP assay of RNA Pol II (D), KLF4 (E) and EP300 (F). *p < 0.05; **p < 0.01; ***p < 0.001.

To determine whether KLF4 alone was necessary to drive the expression of the miR-182 cluster in melanoma, two different shRNAs against KLF4 were transduced into melanoma cells resulting in a clear reduction of endogenous miR-182 cluster levels (Figure 2A–2B). In an attempt to ascertain whether KLF4 directly binds to the miR-182 cluster promoter, chromatin immuno-precipitation (ChIP) experiments were performed. Bioinformatic algorithms predict up to ten KLF4 binding sites in the promoter of the miR-182 cluster. Based on our luciferase reporter results, four different primer sets were designed to cover the KLF4 binding sites with the highest (primer sets #2 and #3) or lowest (primer sets #1 and #4) binding probability (Figure 2C). ChIP-qPCR experiments demonstrated that the RNA polymerase II binds the miR-182 cluster promoter in melanoma cells that do express the cluster, preferentially around the TSS, but not in differentiated cells like the immortal melanocytes Hermes 2B (Figure 2D). ChIP confirmed KLF4 binding to the miR-182 cluster promoter in melanoma cells expressing this cluster (i.e. A375 and SK-MEL-147). Moreover, we found KLF4 preferentially bound to its consensus binding sites located around the TSS (Figure 2E). It has been reported that KLF4 needs to be acetylated for its trans-activation activity, and that acetylation is mediated by the P300/CBP complex [12]. Indeed, we found P300 co-localizing with KLF4 in the miR-182 cluster promoter region (Figure 2F). In summary, these results demonstrate that KLF4 binds to the miR-182 cluster promoter and is necessary to drive miR-182 cluster expression in melanoma cells.

### KLF4 is a physiological regulator of the miR-182 cluster

**Figure 3:**
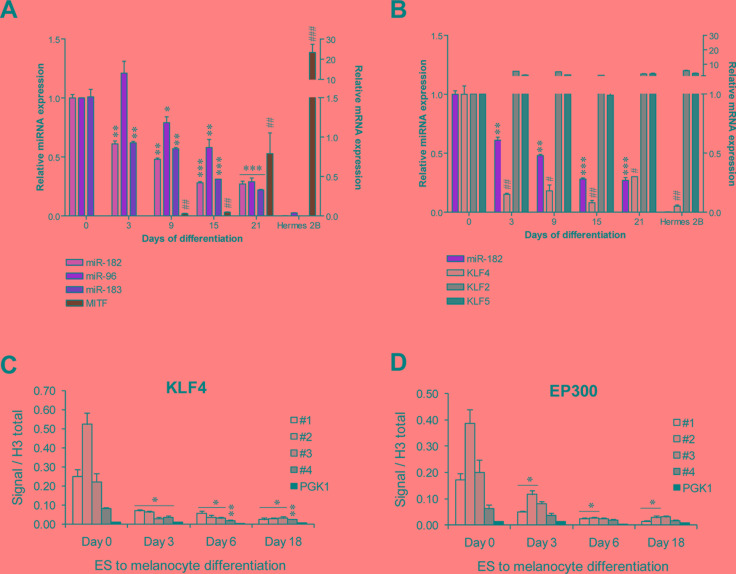
KLF4 regulates the miR-182 cluster expression in hES cells (**A**–**B**) Expression levels of the indicated genes during a hESCs to melanocyte differentiation time course measured by qPCR (*n* = 3/ condition). (**C**–**D**) ChIP assay of KLF4 and EP300 during hESCs to melanocyte differentiation protocol (*n* = 3/ condition). * or ^#^*p* < 0.05; ** or ^##^*p* < 0.01; *** or ^###^*p* < 0.001.

KLF4 is a key regulator of pluripotency and self-renewal capacity of human embryonic stem cells (hESCs) [13]. Since the miR-182 cluster has been shown to be abundantly expressed in mouse and hESCs [10], we investigated whether KLF4 could also be responsible for this miRNA cluster expression in that physiological context. We found that miR-182 cluster is highly expressed in hESCs and progressively repressed during differentiation towards melanocytes, paralleling the increase in melanocyte differentiation markers, such as MITF-M (Figure 3A). We confirmed that miR-182 suppression was not just a general consequence of a defect in the miRNA processing machinery, as levels of DICER1, DROSHA and DGCR8 showed no correlation with the miR-182 cluster levels during the hESCs to melanocyte differentiation time-course (Supplementary Figure 1). However, a direct association between miR-182 and KLF4 levels was observed during the differentiation time course. As a control, other KLF family members, KLF2 and KLF5, were found stably expressed over the differentiation period (Figure 3B). Again, ChIP analysis showed KLF4 occupancy of the miRNA cluster promoter in hESCs cells and progressive loss of binding during melanocytic differentiation (Figure 3C). A parallel pattern was observed for P300 (Figure 3D). Concurring with our results, data mining of ChIPseq analysis of mouse ES cells revealed KLF4 occupancy of the miR-182 cluster promoter (Supplementary Table 3) [14].

### miR-182 is induced by HDAC inhibitor but not by DNA methyl-transferase inhibitor treatment

**Figure 4:**
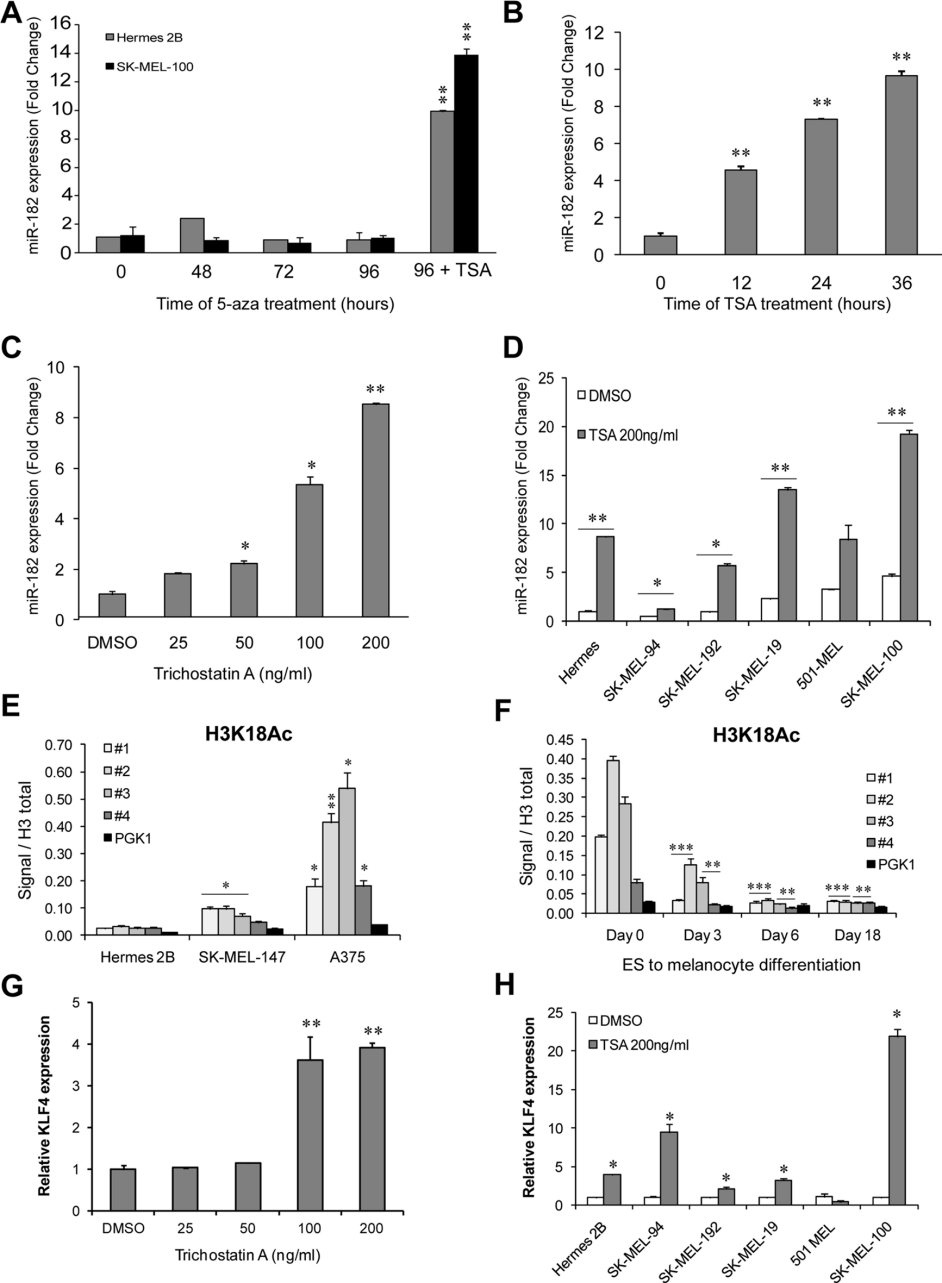
miR-182 is re-expressed upon histone deacetylase inhibition (**A**) MiR-182 expression levels measured by qPCR in immortal melanocytes (grey bars) and melanoma cells (black bars) treated with the DNA methyltransferase inhibitor 5′-azacytidine for the indicated times. The histone deacetylase inhibitor Trichostatin A (TSA) was added for 24 h only at the 96 h time-point of 5-aza treatment. (**B**) Immortal melanocytes treated with 200 ng/ml of TSA for the indicated times. MiR-182 levels were measured by qPCR. (**C**) MiR-182 expression in immortal melanocytes treated with the indicated amounts of TSA during 24 h. (**D**) MiR-182 levels were measured by qPCR in the indicated melanoma cell lines treated with 200 ng/ml of TSA for 24 h. (**E**) ChIP assay of activating histone acetylation marks (H3K18Ac) at the miR-182 cluster promoter in melanoma cell lines and in immortal melanocytes, and (**F**) during hESC to melanocyte differentiation time course. (**G**) KLF4 levels measured in immortal melanocytes treated with the indicated doses of TSA for 24 h. (**H**) Normal immortal melanocytes (Hermes) and melanoma cell lines treated with 200 ng/ml of TSA for 24 h. *p < 0.05; **p < 0.01; ***p < 0.001.

To expand our characterization of the miR-182 cluster's regulation in the melanocytic lineage, we examined whether epigenetic mechanisms could be responsible for the regulation of the miR-182 cluster in differentiated hESCs and melanoma cells in which this cluster is very lowly expressed or undetectable. Treatment with the DNA methyltransferase inhibitor 5′-azacytidine was unable to induce miR-182 levels on immortal melanocytes or melanoma cells lacking miR-182 expression (Figure 4A), suggesting that promoter DNA methylation does not account for this cluster silencing during differentiation or in those cells. However, treatment of immortal melanocytes with the HDAC inhibitor Trichostatin A (TSA) resulted in miR-182 upregulation in a time (Figure 4B) and dose-dependent manner (Figure 4C). The same effect was also observed in melanoma cell lines harboring low miR-182 levels (Figure 4D). Of note, increased histone acetylation levels were found in the cluster promoter on melanoma cell lines that express the cluster compared to immortal melanocytes (Figure 4E). Moreover, histone acetylation at the promoter also decreases during hES cells differentiation to melanocytes (Figure 4F).

Interestingly, it has been reported that KLF4 levels are regulated by histone deacetylase inhibitors (HDACi) in colon cancer cell lines [15] so we tested if in melanoma cells, HDACi treatment was able to induce KLF4 expression. Treatment of melanocytes with TSA resulted in KLF4 upregulation paralleling miR-182 cluster induction (Figure 4G). The same effect was observed in 4 out of 5 melanoma cell lines (Figure 4H).

Overall, our results support a two-step model of miR-182 cluster regulation consisting of histone acetylation relaxing the chromatin structure and thus permitting physical access of TFs, such as KLF4, to the promoter region. During normal differentiation, loss of histone acetylation and KLF4 expression (among other regulatory TFs) might lead to silencing of the cluster. Aberrant expression of the miR-182 cluster in melanoma cells may be due to enhanced histone acetylation of the promoter region together with altered expression of necessary TFs (see Figure 6D).

### KLF4 is essential for melanoma migration, invasion and growth

**Figure 5:**
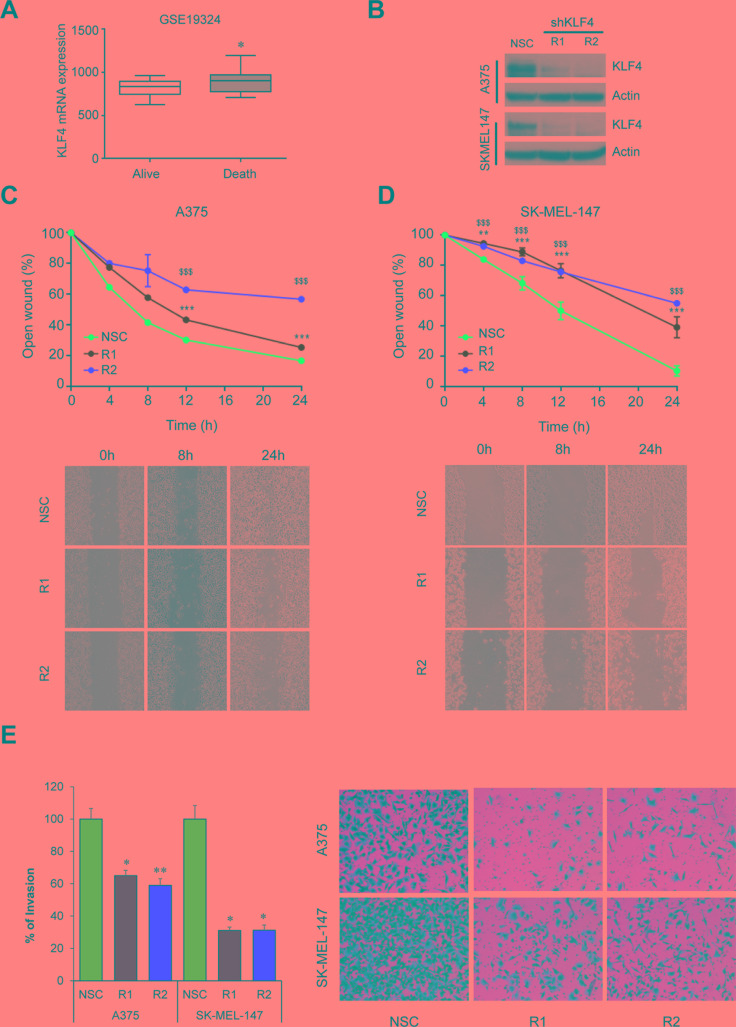
KLF4 silencing impairs migration and invasion (**A**) KLF4 mRNA expression in metastatic melanoma samples (GSE19324, *n* = 44). (**B**) Western blot showing shRNA-mediated KLF4 efficiency in the indicated transduced melanoma cell lines. (**C**) Wound-healing assay on A375 or SK-MEL-147 (**D**) stably transduced with either non-silencing control (NSC) or two different shRNA against KLF4 (R1,R2). Pictures were taken at the indicated times. Graph represents the area of the remaining open wound calculated in relation to time 0 separation (*n* = 6). (**E**) Transwell cell invasion assay in melanoma cells infected with control (NSC) and shKLF4 (R1 and R2) lentiviral particles. Invaded cells were stained with crystal violet (*n* = 3). *p < 0.05; **p < 0.01; *** or ^$$$^*p* < 0.01.

**Figure 6:**
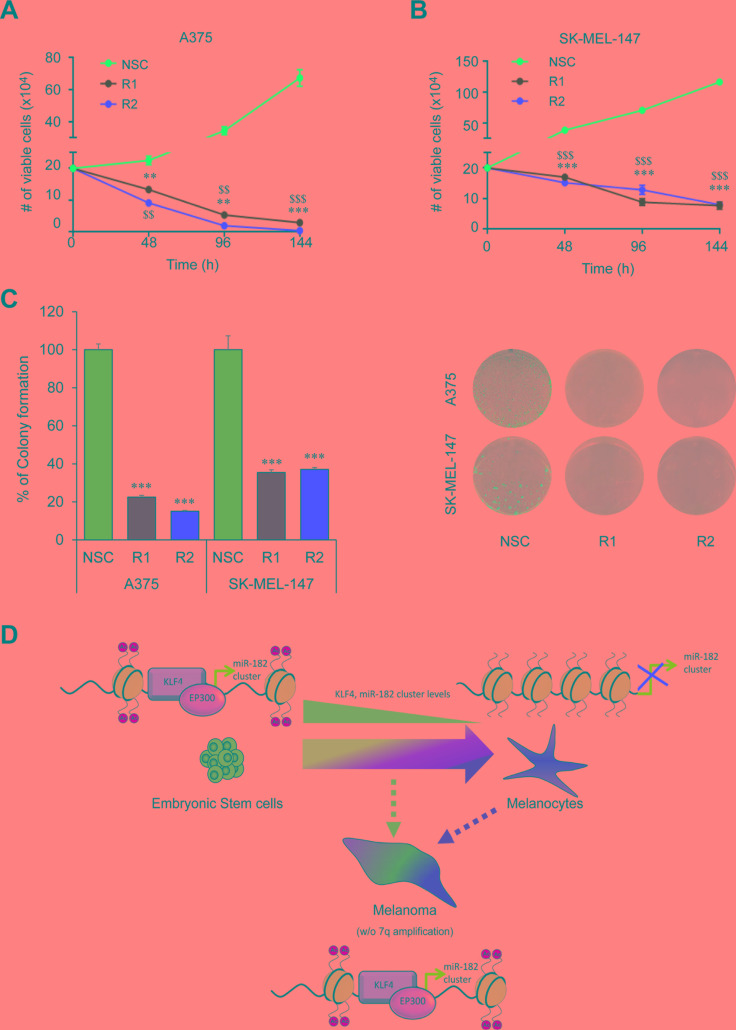
KLF4 silencing reduces cell viability (**A**–**B**) Cell Viability assay in melanoma cells infected with control (NSC) and shKLF4 (R1 and R2) lentiviral particles. Number of viable cells was scored with an automatic cell counter every 48 h (*n* = 3). (**C**) Colony formation assay in shNSC, shKLF4 R1 and shKLF4 R2 infected melanoma cells (*n* = 3). (**D**) Schematic representation of the KLF4-miR-182 cluster regulation under physiological and pathological situations. ** or ^$$^*p* < 0.01; *** or ^$$$^*p* < 0.01.

Several reports support a pro-metastatic role of the miR-182 cluster by promoting migration, invasion and survival of tumor cells in a number of malignancies including melanoma [7]. Therefore, we sought to analyze whether KLF4 could be regulating similar processes and potentially be a new therapeutic target in melanoma. In fact, gene expression analyses in metastatic melanoma samples (GSE19324, *n* = 44) showed higher levels of KLF4 mRNA in patients with poor outcome (Figure 5A) suggesting that KLF4 could contribute to melanoma metastatic progression. Concurring with this hypothesis, shRNA mediated knockdown of KLF4 in melanoma cells (Figure 5B) reduced their ability to migrate in a wound-healing assay (Figure 5C–5D) and to invade in a transwell cell invasion assay (Figure 5E). Furthermore, a drastic reduction in cell viability and colony formation capacity was observed upon shRNA-mediated silencing of KLF4 at longer time points (Figure 6A–6C). These data suggest that KLF4 confers increased survival and aggressiveness to melanoma cells. However, the overexpression of miR-182 was not able to rescue the effects of KLF4 silencing (data not shown), thereby suggesting that other KLF4 targets may also be relevant to KLF4 pro-survival effects in melanoma.

## DISCUSSION

The regulation of miRNA expression is complex and remains undetermined for most miRNAs. In the present study, we focus on the regulation of the miR-182 cluster expression, shown to be deregulated in multiples pathologies [8] and functionally contributing to development of sensory organs and progression of different tumor types such as melanoma (reviewed in [16]).

The miR-182 cluster gene is a large intergenic miRNA gene located in the reverse strand of the long arm of the human chromosome 7 (7q32.2). The primary transcript encodes three different miRNAs (i.e. miR-183, miR-96 and miR-182) which are highly homologous in their seed sequence, especially in the case of miR-96 and miR-182, thereby suggesting a significant number of potentially shared targets.

The first suggestion of a physiological role of this cluster was reported by Xu and colleagues who found miR-182 cluster levels upregulated during the retina development [17]. Moreover, loss of function experiments in zebrafish demonstrated that the miR-182 cluster is necessary for the proper development of sensory epithelia in the inner ear [18].

In addition to its physiological role, aberrant expression of the miR-182 cluster or its components has been reported in several diseases including auto-immune disorders, neuronal and psychiatric disorders and in multiple tumor types (reviewed in [8]). In cancer, most of the reports showed a pro-oncogenic role (e.g. prostate cancer [19], melanoma [7], ovarian cancer [20]) although tumor suppressor roles have also been proposed (e.g. osteosarcoma [21]), suggesting context-dependent functions.

Despite being a highly relevant cluster, very little information exists on the regulation of its expression under physiological and pathological conditions. Chiang et al. found β-catenin/TCF4 binding sites in the promoter of miR-182 cluster and showed that the genetic or pharmacological inhibition of β-catenin impacted negatively on miR-182 levels in breast cancer cell lines [22]. Concurring with their results, the WNT/β-catenin pathway was also shown to be relevant for miR-182 cluster expression in gastric [23] and hepatocellular carcinomas [24]. In melanoma, the WNT pathway may elicit different responses depending on whether the canonical or non-canonical pathway is activated. Recent evidence support that the canonical WNT pathway may be involved in the transformation and proliferation of melanoma cells whereas the switch to the non-canonical WNT pathway may be more relevant to invasion and metastasis [25]. Our *in silico* analysis revealed several WNT-related TF binding sites (i.e. TCF/LEF) but these TFs were not further investigated owing to the lack of correlation with miR-182 levels in melanoma cell lines without 7q genomic locus amplification. We cannot rule out the possibility that the WNT pathway may also contribute to miR-182 cluster expression in melanomas with different genetic background or molecular contexts. Another set of reports propose that the growth factor TGF-β regulates the expression of the miR-182 cluster in glioma [26], gallbladder cancer metastasis [27], prostate cancer bone metastasis [28] and breast cancer [29] but the specific mediators of TGFβ effects were not identified. In melanoma, TGF-β has also been shown to exert tumor promoting functions boosting cell motility and invasiveness [30]. Our promoter analysis revealed two bindings sites (1 also conserved in mouse) for SMAD3, a well-characterized effector of the TGF-β pathway. However, siRNA-mediated knockdown of SMAD3 did not produce alterations in miR-182 levels in melanoma cell lines (Supplementary Figure 2).

Recently, the Kruppel like factor-3 (KLF3) has been shown to repress the expression of miR-182 in soft tissue sarcomas (STS) [31] where miR-182 has a pro-metastatic role [32]. Sachdeva et al. focused on KLF3, KLF11 and KLF15 since are the KLF factors with higher expression in skeletal muscle. Concurring with their results, we also did find several KLF binding sites in the miR-182 cluster promoter. Among them, KLF4 and KLF10 where those TFs that best correlate with miR-182 in melanoma cell lines. However, our experimental validation determined that only KLF4 is bound to the promoter region of the miR-182 cluster in physiologic conditions (hESCs) and in a subset of melanoma cell lines.

In the cancer context, KLF4 has mostly been described as a tumor suppressor. However, there is growing evidence that KLF4 may also behave as an oncogene or a pro-metastatic factor, properties frequently attributed to the miR-182 cluster. For example, KLF4 expression and nuclear localization has been found associated with an aggressive phenotype in early breast cancer [33] and part of a stem cell-like signature in poorly differentiated aggressive human tumors [34]. Furthermore, KLF4 mediates resistance to therapy (i.e. lapatinib) [35] and is required for the maintenance of breast cancer stem cells and for migration and invasion [36]. In prostate cancer, where the miR-182 cluster also is highly expressed [37], indicative of poor prognosis [38] and drives proliferation and invasion [39], KLF4 has been reported to contribute to the progression and have a key role on migration and proliferation of prostate cancer cells *in vitro* and *in vivo* [40]. With all these evidence, it is tempting to speculate that some of the pro-metastatic roles of KLF4 might be attributed to the miR-182 cluster.

In addition to changing tumor cell intrinsic properties, the KLF4-miR-182 cluster axis could contribute to tumor progression by altering the metastatic niche. All members of the miR-182 cluster have been found in exosomes fractions (Exocarta, [41]) and thus may be transferred from stromal tissue to tumor cells as it have been described for other pro-oncogenic miRNAs [42]. Interestingly, high KLF4 levels in normal tissues surrounding colorectal tumors were found indicative of poor prognosis [43] suggesting that some KLF4 downstream effectors, such as the miR-182 cluster, maybe transferred from normal to tumor cells, thereby contributing to tumor genesis or progression.

As most TFs, KLF factors have been considered traditionally ‘undruggable’, but a new class of small molecules able to block the interaction between KLF10 and DNA has been recently developed [44]. These compounds bind to the DNA binding zing-finger domain, a structure common to all KLF members. Therefore, the development of new compounds against other KLF members is now foreseen as feasible. Besides the direct pharmacologic approach, KLF4 activity maybe modulated indirectly. On the one hand, KLF4 needs to be acetylated for its trans-activation activity [14] thus treatment with small molecule inhibitors of histone acetyl-transferases (e.g. P300, CBP) should result in reduced levels of KLF4 targets. In this regard, P300 inhibition in primary melanoma cells suppressed cell proliferation and enhanced cell death when combined with chemotherapeutic agents such as cisplatin [45]. On the other hand, KLF4 expression levels can be modulated with targeted short interference RNAs (siRNAs) or miRNAs. In fact, KLF4 is a reported target of miR-10b in several contexts such as esophageal cancer [46], bladder cancer [47] or gastric carcinoma [48]. Interestingly, miR-10b was reported to be one of the most downregulated miRNAs in melanoma [49], suggesting that miR-10b restoration could have a therapeutic impact in melanoma through the downregulation of KLF4.

In summary, our findings reveal KLF4 as a key regulator of miR-182 cluster expression in hESCs and a main contributor to its aberrant expression in melanoma and potentially in other tumors, thereby providing potential new avenues for therapeutic intervention.

## MATERIALS AND METHODS

### Cell lines and reagents

SK-MEL-19, -94, -103, -147, -187 and -192 melanoma cell lines were kindly provided by Alan Houghton (Memorial Sloan–Kettering Cancer Center) and Hermes cells by Dorothy Bennett (University College London); 501mel were obtained from Yale University. HEK293T and A375 cells were acquired from the American Type Culture Collection. The B16F10 Human melanocytes were purchased from Lonza (adult and neonatal) and Yale University. Melanocytes, Hermes, and SK-MEL and WM cell lines were cultured as described [50]. Human embryonic stem cells were obtained from WiCell and maintained in co-culture with MITC-treated MEFs in the presence of FGF (10 ng/ml; R&D) under conditions described by the supplier. All cultures were maintained at 37°C in a saturated atmosphere of 95% air and 5% CO2. 5-aza-2′-deoxycytidine (5-aza) and trichostatin A (TSA) were purchased from Sigma.

### Plasmids

The pCMV-SPORT6-KLF10 and pCMV-SPORT6-KLF4 constructs were purchased from Open Biosystems. The vectors pCMV6-XL4-BACH1, pCMV6-XL5-PAX9, pCMV6-XL5-ZNF148, pCMV6-XL5-ZNF83, pCMV6-AC-CEBPB, and pCMV6-XL-ZEB1 were purchased from Origene (Rockville, MD, USA). For transactivation experiments, transfection efficiency (70–75%) was monitored by scoring GFP positive cells at 48 hours post-transfection.

For miR-182 cluster promoter analysis, the different indicated constructs were generated by PCR amplification from genomic DNA and cloned into the pGL4-luciferase reporter vector (Promega).

### Microarray expression analysis

The expression levels of the TFs of interest were analyzed from a gene expression dataset of 20 melanoma cell lines previously published by our group (GSE22301), [51]. Array expression values were median centered and log2 transformed. Heatmaps were generated using the MultiExperiment Viewer software (Boston, MA, USA). Gene expression data of melanoma patients (GSE19324, *n* = 44) was used to analyze KLF4 expression in metastatic melanoma samples.

### Luciferase assay

HEK293T or A375 melanoma cell lines were seeded into 96-well plates and co-transfected with 100 ng of the indicated promoter luciferase reporter constructs and 100 ng of the indicated TFs or empty vector (pCDNA3). Luciferase activity was measured using the Dual-Glo Luciferase Assay System (Promega). Renilla luciferase activity was normalized to corresponding firefly luciferase activity and plotted as a percentage of the control.

### Lentiviral production and KLF4 knockdown

pLKO-NSC, pLKO-shKLF4-R1 and pLKO-shKLF4-R2 were purchased from Sigma-Aldrich (Madrid, Spain). Lentiviruses were propagated using previously described methods [52]. Melanoma cells were transduced with viral supernatant and selected with 2 μg/ml Puromycin for 48 h. KLF4 knockdown efficiency was monitored by western blot.

### Real-Time quantitative PCR

Total RNA was extracted using the miRNAeasy Mini Kit (Qiagen). For microRNA expression analysis, quantitative real-time PCR (qRT-PCR) analysis of mir-182, -96, -183, was performed by using miRNA-specific TaqMan MicroRNA Assay Kit (Applied Biosystems); Briefly, 12.5 ng of total RNA were reversed transcribed using the corresponding RT Primer and the TaqMan MicroRNA Reverse Transcription Kit (Applied Biosystems). PCR was performed on 1.33 μl of RT products by adding the TaqMan PCR primers and the TaqMan Universal PCR Master Mix (Applied Biosystems). U6 and RNU44 small RNAs were used for normalization. For mRNA analysis, 1 μg of total RNA was then subjected to DNase treatment and retrotranscription using the High-Capacity cDNA reverse transcription kit (Applied Biosystems, Alcobendas, Spain). Real-time PCR of KLF2, KLF4, KLF5 and MITF genes was performed using SYBR green fluorescence (Applied Biosystems). GAPDH was used as an internal standard. Primers sequences are listed in Supplementary Table 4. Relative quantification of gene expression was performed with the 2^(−ΔΔCt)^ method [53].

### Western blot

Cell lysates were obtained in RIPA buffer (Pierce, Thermo Scientific), supplemented with 1x EDTA-free complete protease inhibitor cocktail (Roche). Protein concentration was quantified by a modified Lowry assay (DC protein assay; Bio-Rad). 30 μg of protein were resolved in NuPAGE 4–12% Bis-Tris gels and transferred to iBlot^®^ Gel Transfer Stacks PVDF membranes (Life Technologies, Thermo Fisher Scientific). After blocking with Tris-buffered saline with Tween-20 containing 5% non-fat dry milk or 5% BSA for 1 h at room temperature, membranes were probed overnight at 4°C with the following antibodies: anti-KLF4 [1:1000, Santa Cruz #sc-20691]; anti-Rabbit IgG-Peroxidase antibody produced in goat [1:10.000, Sigma-Aldrich #A0545] and anti-Actin HRP [1:40.000, Santa Cruz #sc-1616]. Membranes were developed with SuperSignal Dura detection kit (Pierce) or EZ-ECL Chemiluminiscence detection kit (Biological Industries, Kibbutz Beit-Haemek, Israel).

### Chromatin immunoprecipitation

Chromatin Immunoprecipitation experiments were performed using the EZ-Magna ChIP™ A (Millipore) following manufacturer's instructions. Briefly, 1 × 10^7^ cells were fixed with 1% formaldehyde and nuclei were isolated and sonicated to generate ∼500-1000 bp DNA fragments. Fragmented chromatin was incubated with 1 μg of RNA pol II, EP300, Histone 3, H3K18Ac, KLF4 or IgG control antibodies (Abcam) and immunoprecipitated with protein A magnetic beads. After washes, protein/DNA immunoprecipitates were eluted and reverse crosslinked with Proteinase K at 62° for 2 h. ChIP and input DNA were quantified by RT-qPCR. Expression values were normalized versus total Histone 3 signal.

### hESC differentiation

H9-hES cells were induced to form Embryo Bodies for 4 days followed by a transfer to differentiation media (0.02 μm Dexamethasone, 1X Insulin-transferrin-selenium, 1 mg/ml linoleic acid-bovine serum albumin, 30% Low Glucose DMEM, 20% MCDB 201, 0.1 mM L-ascorbic-acid, 50% Wnt3-Conditioned media, 50 ng/ml hSCF, 100 nM EDN3, 20pM cholera toxin, 50 nM TPA, 4 ng/ml bFGF) and seeded in 6 replicates. Media was changed daily and cells were collected after 0, 3, 9, 15 and 21 days of differentiation.

### Wound-healing migration assay

An artificial “wound” was created on a confluent cell monolayer (2 × 10^6^ cells in 6-well plates) of A375 and SK-MEL-147 melanoma cells previously transduced with pLKO-NSC or shKLF4-R1 and R2 lentiviral vectors. Six preset fields per condition were photographed at the indicated times. The migration capability of melanoma cells was calculated as a percentage of wound healing vs. time 0. The wound area was calculated using Image J software (National Institutes of Health, Bethesda, MD, USA).

### Transwell cell-invasion assay

A suspension of 2 × 10^5^ A375 and 1.5 × 10^5^ SK-MEL-147 melanoma cells previously transduced with shRNAs (NSC, R1 and R2) was added in serum-free media to matrigel-coated (0.87 mg/ml) 8-μm-pore cell culture inserts (Falcon Discovery Labware; BD Biosciences). Cells were incubated for 24 h under standard culture conditions and complete media in the lower part of the chamber. Viable cells that had migrated to the lower transwell chamber were stained with crystal violet. Crystals were dissolved in 15% acetic acid and the absorbance was measured at 590 nm.

### Cell viability and colony formation

For cell viability assays, pLKO-NSC or shKLF4-R1 and R2 transduced melanoma cells were seeded at 200,000 cells into 35 mm-dish with counted and reseeded at days 2, 4 and 6 (*n* = 3/condition). For colony formation assays, pLKO-NSC or shKLF4-R1 and R2 infected melanoma cells were seeded at 2,500 cells/well in 6-well plates in triplicates with 0.5 μg/mL of puromycin. Medium was refreshed every 3 days and cells were allowed to grow for 10–15 days. Then, cells were stained with crystal violet. Crystals were dissolved in 15% acetic acid and the absorbance was measured at 590 nm.

### Statistical methodologies

Statistical significance was determined by unpaired *t*-test (GraphPad Prism Software) and McNemar's test for correlations of expression data.

## SUPPLEMENTARY MATERIALS FIGURES AND TABLES


