# Supplementary material for: Krüppel-like factor 4 (KLF4) regulates the miR-183~96~182 cluster under physiologic and pathologic conditions
Source: Oncotarget. 2017 Feb 17;8(16):26298–311. doi: 10.18632/oncotarget.15459 (PMC5432258; doi:10.18632/oncotarget.15459)
Supplement: Supplementary file 1 [file oncotarget-08-26298-s001.pdf]

Krüppel-like factor 4 (KLF4) regulates the miR-183~96~182 cluster under physiologic and pathologic conditions

Supplementary Materials

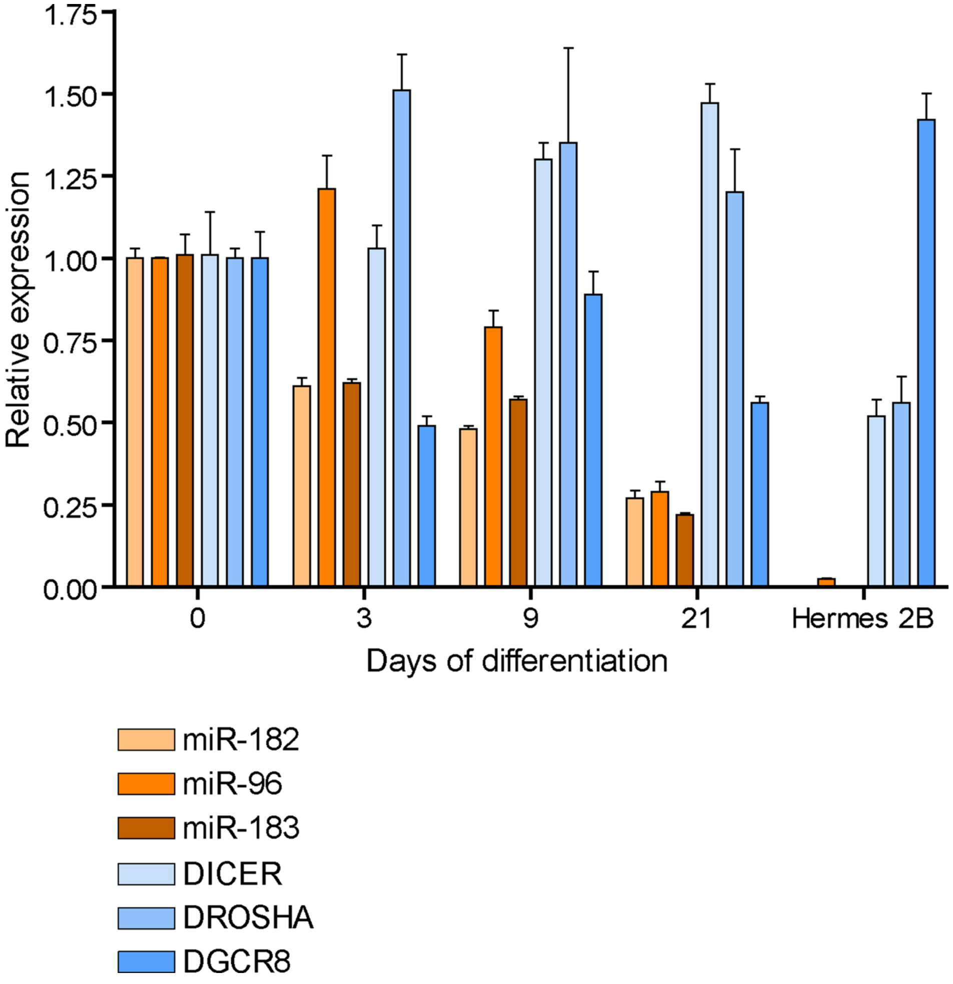

Supplementary Figure 1: miRNA processing machineri component expression is not altered during hESC differentiation to melanocytes. mRNA levels of DROSHA, DICER and DGCR8 were measured by qPCR in a hESCs to melanocyte differentiation time course. The levels of mature forms of the miR-183, miR-96 and miR-182 were also analyzed by qPCR. Expression values were normalized versus normal melanocytes.

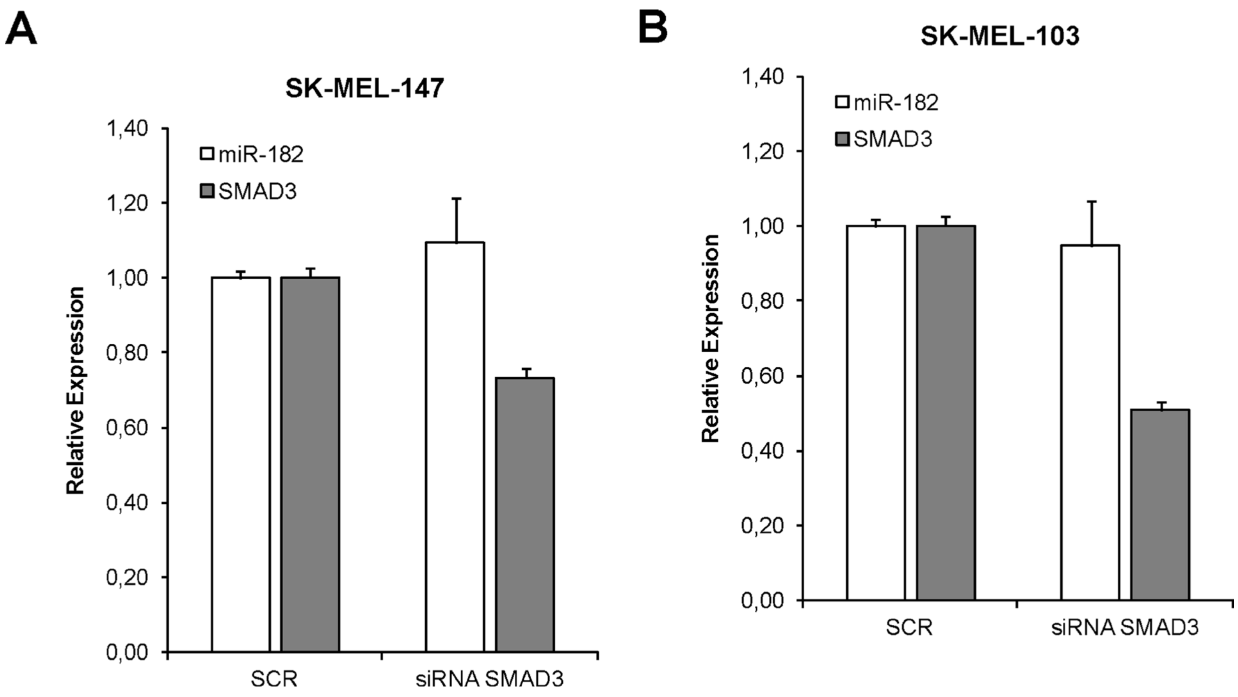

Supplementary Figure 2: SMAD3 does not regulate miR-182 levels in melanoma. Relative expression levels of miR-182 and SMAD3 analyzed by qPCR after transfection of SK-MEL-147 (**A**) or SK-MEL-103 (**B**) melanoma cell lines with siRNA scrambled or siRNA against SMAD3.

Supplementary Table 1: TFs present in the 0.8 Kb region upstream the miR-183~96~182 cluster promoter TSS

Transcription Factor	Strand	Matrix sim.	Core sim.	Sequence	# Sites in human	# Sites in Mouse
SP1	–	0.985	1	gggGGGAggggaagt	12	5
ZBP89 (ZNF148)	+	0.952	1	cggctcccctCCCCctccctctc	5	6
ZNF219	–	0.971	1	ggagccgCCCCcagacccgttcc	5	6
MAZ	–	0.986	1	gaggGAGGggggag	9	5
CTCF	–	0.913	0.958	gcagggggcgggccGGGGatgattcac	8	4
CKROX	–	0.973	1	agggGGGAggggaagtg	6	4
KLF6	–	0.945	0.958	ggcaGGGGgcgggccgg	5	4
EGR1	–	0.831	0.75	ggctgcgcgGGGGctcg	4	4
KKLF	+	0.989	1	gggggagGGGGagatgg	8	3
WT1	–	0.942	1	ctgggAGGGgggaggggg	7	3
HIC1	–	0.85	0.75	cgggcTGCCcgga	3	3
E2F	+	0.771	0.78	ggggggcggTAAAaccc	2	3
MZF1	–	0.946	1	gaGGGGagccg	7	2
PLAG1	+	0.985	1	GAGGgcaggaggcggggatag	6	2
GC	–	0.992	1	agagGGAGgggggagg	3	2
AP1	+	0.882	0.961	ggtgAATCatc	2	2
XCPE1	+	0.865	0.811	ggGCGGggccc	2	2
PAX5	–	0.887	1	ctggggCGCAccgtagggccactggacga	2	2
RREB1	+	0.888	1	cCCCAttccagccct	2	2
VDR_RXR	+	0.955	1	gagggtgggcgtgtGAGGacatgga	2	2
NRSE	+	0.938	1	ccgggcgcacctCGGCtagcggcaccctagc	3	2
AHRARNT	+	0.994	1	ccgagggtggGCGTgtgaggacatg	4	1
CDE	–	0.879	0.75	ggggCGCGggctg	3	1
PAX9	+	0.918	1	ggCACCctagcccgagggcag	3	1
PURALPHA	–	0.903	1	ggAGGGggaggggg	3	1
AP2	+	0.911	0.807	gggGCCCggggcggg	2	1
ARE	–	0.977	1	aggggcctccatGTCCtca	2	1
EKLF	+	0.924	1	cctcccaGGGTcggatt	2	1
GRHL1	–	0.892	1	ggctccGGTTggt	2	1
OLF1	–	0.91	1	tgtctgTCCCaagagagggaggg	2	1
SMAD3	–	0.928	1	tGTCTggcc	2	1
TIEG (KLF10)	–	0.997	1	gaGGTGtgtgtggcg	2	1
ZNF300	+	0.952	1	ctacggtgcgCCCCagctcccaa	2	1
AP4	+	0.996	1	ggcggCAGCggtggcca	1	2
AREB6 (ZEB1)	+	0.985	1	cagaCACCtgtga	1	2
ATF6	–	0.877	1	gtggcgtGGCGtgcttggaaa	1	2
BACH1	+	0.898	0.959	tcaaggTGAAtcatccccggc	1	1
BKLF	+	0.958	1	cgaGGGTgggcgtgtga	1	1
CDP	+	0.893	0.833	ctgagcCAATggcgctgcg	1	1
CEBPB	+	0.973	1	cgggttggGCAAgca	1	1
CMYB	+	0.958	1	acCAACcggagcc	1	1
DEC2	+	0.797	0.844	gcggcaCCTGagcca	1	1
E2F1_DP2	+	0.797	1	agaaGGCGggcgacggg	1	1
FXRE	+	0.839	0.96	AGGTgaatcatcc	1	1
KLF4	–	0.743	1	aagagagggAGGGggag	1	1
GLI3	–	0.951	1	acgcCCACcctcggc	1	1
GLIS3	–	0.831	0.789	accgCCCCccgcccc	1	1
HBP1	–	0.836	0.765	gtgcacagagAATGactccagga	1	1
HNF4	–	0.961	1	ccgccgggaaCAAAggctgtctgtc	1	1
HOXB9	+	0.902	0.778	gggggcggTAAAaccct	1	1
HPF1 (ZNF83)	–	0.87	1	ggaatggggacAAGGggcctccatg	1	1
IRF4	–	0.859	0.767	cacaGAGAatgactccaggag	1	1
LEF1	–	0.97	1	ccgggaaCAAAggctgt	1	1
MIF1	–	0.923	1	acaggtgtctgGCCAccgc	1	1
MOK2	+	0.903	1	tcagccttgagcaCCTTtctg	1	1
MYCMAX.03	+	0.783	0.75	cagccgCGCGgtc	1	1
NF1	+	0.991	1	cccTTGTccccattccagccc	1	1
NFKAPPAB50	–	0.863	1	gccGGGAgccccg	1	1

Supplementary Table 2: TFs with a positive correlation with miR-182 levels in melanoma cell lines without 7q amplification.

Gene	Correlation	Human Sites	Mouse Sites
SMAD3	0.89	2	1
BACH1	0.86	1	1
PAX9	0.84	3	1
HPF1	0.83	1	1
CEBPB	0.80	1	1
ATF6	0.78	1	2
RREB1	0.76	2	2
ZBP89	0.74	5	6
KLF10	0.69	2	1
NF1	0.68	1	1
KLF4	0.66	1	1
CUX1	0.59	1	1
ZEB1	0.58	1	2
KLF3	0.57	1	1
GC	0.57	3	2
ZNF219	0.52	5	6

Supplementary Table 3: Coordinates of loci bound by transcription factors in the miR-182 cluster promoter in mouse ES cells

Transcription Factor	Coordinates	CpG island
Esrrb	chr6:30121639-30121639<br>chr6:30124289-30124291	No<br>Yes
Ctcf	chr6:30123282-30123283	No
E2f1	chr6:30121816-30121831<br>chr6:30123098-30123105<br>chr6:30124431-30124433<br>chr6:30125543-30125544	No<br>Yes<br>Yes<br>No
Tcfcp2l1	chr6:30123083-30123094<br>chr6:30124349-30124383<br>chr6:30125867-30125867	Yes<br>Yes<br>No
Zfx	chr6:30124660-30124663<br>chr6:30125631-30125631	No<br>No
Klf4	chr6:30121414-30121420<br>chr6:30123218-30123226<br>chr6:30124307-30124313<br>chr6:30124815-30124885<br>chr6:30125811-30125845	No<br>Yes<br>Yes<br>No<br>No

miR-182 cluster locus: chr6:30120000-30126000.

Supplementary Table 4: Primers used for qPCR analysis

Gene symbol		Sequence (5′ to 3′)
KLF2	Fw:<br>Rv:	CACCAAGAGTTCGCATCTGA<br>GGCTACATGTGCCGTTTCAT
KLF5	Fw:<br>Rv:	CCTGGTCCAGACAAGATGTG<br>GTCAGGGAGGAAGACGTTCA
KLF4	Fw:<br>Rv:	ACACGAAAAGCTCCCATTTG<br>TGTCCGGTGTGTTTCCTGTA
MITF-M	Fw:<br>Rv:	CTACCGTCTCTCACTGGATTGGTGC<br>GCTTGCTGTATGTGGTACTTGGTGG
GAPDH	Fw:<br>Rv:	CGCTCTCTGCTCCTCCTGTT<br>CCATGGTGTCTGAGCGATGT
